# Experimental Study of Cement - Sandstone/Shale - Brine - CO_2 _Interactions

**DOI:** 10.1186/1467-4866-12-9

**Published:** 2011-11-11

**Authors:** Susan A Carroll, Walt W McNab, Sharon C Torres

**Affiliations:** 1 Lawrence Livermore National Laboratory, 7000 East Avenue, Livermore CA 94550, USA

## Abstract

**Background:**

Reactive-transport simulation is a tool that is being used to estimate long-term trapping of CO_2_, and wellbore and cap rock integrity for geologic CO_2 _storage. We reacted end member components of a heterolithic sandstone and shale unit that forms the upper section of the In Salah Gas Project carbon storage reservoir in Krechba, Algeria with supercritical CO_2_, brine, and with/without cement at reservoir conditions to develop experimentally constrained geochemical models for use in reactive transport simulations.

**Results:**

We observe marked changes in solution composition when CO_2 _reacted with cement, sandstone, and shale components at reservoir conditions. The geochemical model for the reaction of sandstone and shale with CO_2 _and brine is a simple one in which albite, chlorite, illite and carbonate minerals partially dissolve and boehmite, smectite, and amorphous silica precipitate. The geochemical model for the wellbore environment is also fairly simple, in which alkaline cements and rock react with CO_2_-rich brines to form an Fe containing calcite, amorphous silica, smectite and boehmite or amorphous Al(OH)_3_.

**Conclusions:**

Our research shows that relatively simple geochemical models can describe the dominant reactions that are likely to occur when CO_2 _is stored in deep saline aquifers sealed with overlying shale cap rocks, as well as the dominant reactions for cement carbonation at the wellbore interface.

## Background

Carbon dioxide is actively being stored at depth in a sandstone saline reservoir as part of the In Salah Gas Project in Krechba, Algeria [1]. It is one of few commercial scale CO_2 _storage projects and serves as an important platform to study the scientific and technical issues for safe and effective long-term CO_2 _storage in deep saline reservoirs [2-11].

Wellbores are a potential risk pathway for leakage of CO_2 _from the storage reservoir to overlying drinking water aquifers and back into the atmosphere. Carbonation of cements, used in wellbores to seal off fluid flow from the reservoir, can bring about changes in permeability and alter the movement of fluids within the wellbore environment. Field, experimental and modeling studies suggest that carbonation of hydrated cements lowers porosity and has the potential to heal fractures within the cement [12-18].

Risk of leakage from a CO_2 _storage reservoir would be significantly reduced if the CO_2 _could be stored as a solid carbonate mineral and if these reactions improved the seal within the cap rock above the reservoir. Field and laboratory experiments have shown mineral dissolution in CO_2_-rich brines leads to increased concentrations of Ca, Fe, and Mg and, in some cases, to the formation of carbonate minerals [19-24]. The amount of CO_2 _stored as carbonate minerals over geologic times estimated from reactive transport simulations varies substantially and depends on the reaction rates and the amount of CO_2 _injected into the subsurface [25-30]. The possibility of even small amounts of carbonate mineralization in shale cap rock may significantly improve seal integrity by reducing porosity. Simulation results suggest seal integrity is enhanced due to carbonate mineral precipitation after 100 years of reaction with CO_2_-rich fluids [31]. Another modeling study predicts that redistribution of calcite within 0.1 m of the cap rock - reservoir interface effectively seals reservoir from the overlying strata [32].

The focus of this work was to determine the key geochemical reactions involving common cements used in wellbore construction, formation mineralogy, and supercritical CO_2 _stored at the Krechba site. We reacted the end member components of the heterolithic sandstone and shale unit that forms the upper section of the carbon storage reservoir with supercritical CO_2 _and representative brine with and without cement at 95°C and 10 MPa in gold bag autoclaves. Separate cement experiments without CO_2 _were conducted to measure cement hydration at temperature prior to the injection of CO_2_. The experimental results can be used to develop geochemical models for estimating long-term trapping of CO_2_, and wellbore and cap rock integrity at the Krechba site.

## Methods

### Materials

The heterolithic sandstone and shale in units C10.2 and C10.3 form the upper section of the carbon storage reservoir at the Krechba Field, In Salah, Algeria. Reported mineralogy among 17 core samples collected from Krechba reservoir ranged from a quartz-dominated sandstone to a shale-like material containing abundant illite clay [33]. Iron-rich chlorite appears as coatings on quartz grains in the sandstone and in abundances as high as 30 percent by volume in the shale. Other aluminosilicates include minor quantities (e.g., typically less than 5 percent) of kaolinite and feldspar. Carbonate phases have been described as siderite [33], ankerite plus dolomite (unpublished mineralogical analyses conducted by Statoil), or calcite (unpublished XRD analyses of Krechba core samples). The shale end member, Sample 14, consisted of 44% illite, 30% chlorite, 20% quartz, 4% kaolinite, 2% feldspar and trace amounts of pyrite by weight. The sandstone end member, Sample 7, consisted of 88% quartz, 6% chlorite, 4% kaolinite and 2% siderite by weight. Limited availability of the heterolithic sandstone and shale necessitated the use of rock fragments in the experiments rather than a well-defined powdered size fraction.

The powdered class G oil well cement used in the experiments was provided by Mountain Cement Company and consists of 56% Ca_3_SiO_5_, 39% Ca_2_SiO_4_, 5% Ca_3_AlO_4.5_, + 0.5% Na_2_O and K_2_O by weight as determined by standard *ASTM C 150*. In some experiments small amounts of bentonite were added to the cement to reflect mixtures identified in well logs from the Krechba site (bentonite to cement ratio = 1:39 by weight). Any curing of the cement occurred in the reaction vessels at the experimental conditions. Combination of powdered cement and rock fragments did not compromise the results because the primary objective of the experiments was to determine the dominant geochemical reactions controlling the solution composition.

Initial solutions were distilled and deionized water, 0.13 m CaCl_2_, and synthetic Krecha brine consisting of 1.8 molal NaCl, 0.55 molal CalCl_2_, and 0.1 molal MgCl_2_. All salts used to synthesize the brines were reagent grade. The experiments were conducted in a synthetic brine to capture the major ion chemistry measured at the site. A more complex reservoir brine was not used to avoid masking relevant geochemical reactions. High purity liquid CO_2 _was pressurized at temperature and pressure to generate supercritical CO_2 _for the experiments.

### Cement Hydration Experiments

Distilled and deionized, 0.13 m CaCl_2_, and synthetic Krechba solutions were used to determine ion activity products for cement hydration at different solid:solution ratios at 115 and 95°C (Table 1). Solutions and solids were reacted in teflon-lined Parr reaction vessels, sealed, and placed into an oven to maintain temperature. Sealed reaction vessels were quenched in cold water prior to taking filtered aqueous samples for chemical analyses. Solids were washed with distilled and deionized water and dried at 60°C prior to analysis by an environmental scanning electron microscopy with energy dispersive x-ray spectroscopy (ESEM/EDX) and powder x-ray diffraction (XRD).

**Table 1:** Cement Hydration Experiments.

ID	Solid	Solution	Solid:Soln (g/g)	T °C	Days	pH(c,25)
G3	A	0.13 m CaCl_2_	1:10	115	43	11.9
G6	A	Brine	1:10	115	43	not measured
G7	B	MQ water	1:10	115	58	12.2
G8	B	MQ water	1:10	115	87	12.3
G9	B	MQ water	2:10	115	58	12.3
G10	B	MQ water	2:10	115	87	12.1
G11	B	MQ water	1:10	95	74	12.3
G12	B	MQ water	2:10	95	43	12.3
G13	B	MQ water	2:10	95	74	12.1
G14	B	Brine	1:10	95	88	11.4
G15	B	Brine	2:10	95	88	12.3

A indicates Class G cement, B indicates Class G cement plus bentonite (39 g cement to 1 g bentonite), and Brine indicates 1.8 molal NaCl, 0.55 molal CaCl_2 _and 0.1 molal MgCl_2 _solution. Note that brine pH values are conditional (c) because of the high ionic strength.

### Cement - Rock - Brine - CO_2 _Experiments

Static Dickson-type Au reactors housed in water-filled pressure vessels were used to react cement, sandstone, shale, synthetic brine and supercritical CO_2 _at 95°C and 10 MPa. Specific weights of cement, sandstone, shale, and brine are listed in Table 2. Coherent sandstone or shale rock fragments were used in the experiments due to limited availability from core. We monitored reaction kinetics and the approach to equilibrium by sampling the solution as a function of time. The reactor setup allows sequential sampling of the aqueous phase while the experiment is at pressure and temperature. All metals measured in solution were from the rock-fluid interactions, because the supercritical CO_2 _and the brine contact only gold or passivated titanium. After one month of reaction, supercritical CO_2 _was injected into the gold bag and reacted for an additional month. About 20 grams of supercritical CO_2 _were added to the reaction vessel to ensure excess CO_2 _during the reaction. To add the CO_2_, liquid CO_2 _was pressurized above the run pressure and injected into the reaction vessel through the sample tube. The liquid CO_2 _transitions to supercritical CO_2 _at the run pressure and temperature. The amount of CO_2 _injected was estimated from change in volume of the liquid CO_2_. Several brine samples were taken and analyzed for solution chemistry over the duration of the experiment. At the end of the experiment, the reaction vessel was cooled to room temperature, excess CO_2 _was removed, and solid reactants were rinsed with distilled and deionized water several times to remove brine. The solids were dried at 60°C prior to XRD and ESEM/EDX analysis. Samples for dissolved Al, Ca, Fe, Mg, and Si analyses were filtered, and directly diluted with acidified distilled and deionized water (using high purity HNO_3_).

**Table 2:** Cement-Rock-Brine-CO_2 _Experiments.

ID	Cement (g)	Shale (g)	Sandstone (g)	Brine (g)	Days reacted before CO_2_	Days reacted after CO_2_
GBCO2_1	4.0			280.5	11	30
GBCO2_2	20.0			204.5	21	22
7CO2			5.6	301.2	33	28
14CO2		6.4		301.2	31	31
GB7CO2	4.8		4.9	252.5	26	44
GB14CO2	8.6	8.7		246.5	40	35

All experiments were conducted in 1.8 molal NaCl, 0.55 molal CaCl_2 _and 0.1 molal MgCl_2 _brine with 20 grams of supercritical CO_2 _at 95°C and 10 MPa.

Samples for total dissolved inorganic carbon were injected directly into 1 N NaOH to trap the CO_2_, filtered to remove any solids that precipitated, and analyzed for dissolved inorganic carbon, calcium, and magnesium. Total dissolved carbon should be equal to the measured inorganic carbon in the filtered sample plus the amount of carbon trapped as calcite minerals in the NaOH extraction. Comparison of results from the NaOH extraction with estimates from Duan and Sun (2003) caused us to question the viability of using the extraction technique to quantify dissolved carbon in the experiments. Although the median value of the dissolved carbon concentrations estimated from the extraction technique (0.78 molal) agrees with the theoretical prediction (0.69 molal), there is a significant amount of scatter in the extracted values over time (ranging from 1.12 to 0.53 molal). The scatter largely correlates to changes in measured dissolved calcium, because calcium is predicted to be trapped as CaCO_3 _solid rather than some mixture of CaCO_3 _+ Ca(OH)_2 _solids (any Mg is predicted to be trapped as Mg(OH)_2 _and was not considered in the NaOH extraction method). We have chosen to use the theoretical dissolved CO_2 _concentrations in the development of the geochemical model because of the uncertainty associated with the NaOH extraction chemistry. However, we report both the extraction and theoretical values, because caustic extractions are commonly used to quantify total dissolved CO_2_.

### Analysis

Major and trace metals in the aqueous samples and the stock solution were analyzed using inductively coupled plasma mass spectroscopy (ICP-MS, Make/Model: Thermo Electron Corp/X Series Q-ICPMS). Samples were prepared volumetrically using an internal standard solution in 2% nitric acid. A fully quantitative analysis using a linear calibration curve based on known standards was performed. The internal standard was corrected for instrument drift and suppression from the sodium chloride matrix. Silica was run in collision cell technology (CCT) mode to avoid polyatomic interferences. Detection levels were established from duplicate blanks and serial dilution preparations. Matrix spike samples were analyzed for quality control. Detection limits were about 3, 0.2, 0.5, 4, and 0.35 ng/g for Ca, Mg, Al, Si, and Fe, respectively.

Total inorganic carbon (TIC) concentrations are determined using an automated OI Analytical Aurora 1030W Carbon Analyzer. The Aurora 1030W uses a syringe pump to transfer samples and reagents to a temperature-controlled reaction chamber. TIC samples are reacted with 5% phosphoric acid to evolve CO_2 _gas purged by a stream of N_2 _gas and quantified using a NDIR detector.

Solid mineralogy was determined from data collected from random orientation powder samples with a Scintag PAD V instrument using a Cu-Kα source at 45 kV and 35 mA from 5° to 70° 2Θ in 0.02° steps. XRD cannot detect amorphous solids or minerals that are present at less than 2 wt%.

Unreacted and reacted samples were analyzed using a Quanta 200 Environmental Scanning Electron Microscope in low vacuum mode with EDX. Images were collected with secondary and backscatter detectors from pressures ranging from 0.23 to 0.90 torr and 20-25 kV. EDX was use to determine the local chemical composition of materials using at 20kV and 11 mm working distance. All analyses are semi-quantitative.

### Geochemical Modeling

Solution compositions from the batch experiments were modeled using the PHREEQC 2.15.0 geochemical code [34], and the SUPCRT92 thermodynamic database [35] augmented by CEMDATA07v2 [36]. The CEMDATA provides Gibbs free energies, heat capacity, and volume data for cement phases as a function of temperature and pressure [37,38]. The standard state for minerals and pure water is unit activity, and for all aqueous species other than dissolved CO_2 _is unit activity in a hypothetical 1 molal solution referenced to infinite dilution at any pressure and temperature. The dissolved CO_2 _concentrations were calculated assuming equilibrium with *f*_CO2 _estimated from CO_2 _equation of state at 10 MPa [39]. No other mass balance reactions were corrected for pressure. This introduces an error of about ± 0.1 log K. pH was estimated from the forward model simulations. The B-dot ion interaction model was used to approximate the non-ideal behavior of solutions at elevated ionic strength and temperature. The B-dot equation is an extended form of the Debye-Huckel equation and was used in this study because it can be applied to NaCl based solutions with high ionic strengths (3 molal) over a wide range of temperature. However, it is generally recognized that the B-dot equation becomes increasing less accurate at I > 0.5 molal. Despite these limitations, we chose to use B-dot equation to correction for species activity because the Pitzer equations are lacking for many elements at temperatures above 25°C. The use of the B-dot equation typically yields brines with slightly higher solution pH (≈ 0.1 pH units). The thermodynamic and kinetic inputs to the geochemical model for reaction of cement, sandstone and shale with CO_2_-rich Na-Ca-Mg chloride brines are shown in Tables 3, 4, 5, 6, and 7.

**Table 3:** Mineral weight percents used in geochemical simulations.

Phase	Sandstone	Shale
Albite	1.5%	2%
Chlorite	6%	30%
Dolomite	0.5%	0.75%
Illite	1.5%	44%
Kaolinite	4%	4%
Quartz	86%	20%
Siderite	1.5%	0.75%

(Ankerite was modeled as a 25 - 75 siderite-dolomite mixture for sandstone and a 50 - 50 siderite-dolomite mixture for the shale.)

**Table 4:** Surface areas used in the modeling calculations.

Phase	Surface Area (cm^2^/g)	
	Shale	Sandstone
Boehmite	0.02	0
Smectite	9505	317.7
Ripidolite	224.2	87.9
Dolomite	0.5	1.0
Illite	9505	317.7
Kaolinite	18.3	17.9
Low-albite	45.3	33.4
Quartz	9.1	39.1
Siderite	0.4	2.2

**Table 5:** Geochemical model for reaction of cement, sandstone and shale with CO_2 _and Na, Ca, Mg chloride brines.

Cement Hydration			
Phase	Mass Balance	Log K 95°C	Log SI 95°C
Portlandite	Ca(OH)_2 _+ 2H^+ ^⇔ Ca^2+ ^+ 2H_2_O	18.30	0.04 ± 0.04
Psuedowollastonite	CaSiO_3_+ 2H^+ ^⇔ SiO_2 _+ Ca^2+ ^+ H_2_O	10.97	0.3 ± 1.3
Brucite	Mg(OH)_2 _+ 2H^+ ^⇔ Mg^2+ ^+ 2H_2_O	12.65	0.7 ± 0.1
^1^Hydrotalcite	Mg_4_Al_2 _O_7_(OH)_2_:10H_2_O + 14H^+ ^⇔ 2Al^3+ ^+ 4Mg^2+ ^+ 17H_2_O	53.67	2.8 ± 0.2
^1^Fe-Hydrogarnet	Ca_3_Fe_2_(OH)_12 _+ 12H^+ ^⇔ 3Ca^2+ ^+ 2Fe^3+ ^+ 12H_2_O	68.50	-3.4 ± 2.7
Anhydrite	CaSO_4 _⇔ Ca^2+ ^+ SO_4_^2-^	-5.08	-0.3 ± 0.1
**Cement, Sandstone, and Shale Carbonation**			
**Phase**	**Mass Balance**		**Log K 95°C**
Albite	NaAlSi_3_O_8 _+ 4H^+ ^⇔ Al^3+ ^+ Na^+ ^+ 2H_2_O + 3SiO_2_		0.46
^1^Amorphous Al(OH)_3_	Al(OH)_3 _+3H^+ ^⇔ Al^3+ ^+ 3H_2_O		5.42
^1^Amorphous Fe(OH) _3_	Fe(OH)_3 _+3H^+ ^⇔ Fe^3+ ^+ 3H_2_O		2.86
Boehmite	AlO(OH) + 3H^+ ^⇔ Al^3+ ^+ 2H_2_O		3.75
Calcite	CaCO_3 _+ H^+ ^⇔ Ca^2+ ^+ HCO_3_^-^		0.85
Chalcedony	SiO_2 _⇔ SiO_2,aq_		-2.88
Dolomite	CaMg(CO_3_)_2 _+ 2H^+ ^⇔ Ca^2+ ^+ Mg^2+ ^+ 2HCO_3_^-^		1.41
Ripedolite 14Å	Mg_3_Fe_2_Al_2_Si_3_O_10_(OH)_8 _+16 H^+ ^⇔ 2Al^3+ ^+ 3SiO_2,aq _+ 3Mg^2+ ^+ 2Fe^2+ ^+ 12H_2_O		41.45
Illite	K _0.6_Mg_0.25 _Al_2.3_Si_3.5_O_10_(OH)_2 _+ 8H^+ ^⇔ 0.25Mg^2+ ^+ 0.6K^+ ^+ 2.3Al^3+ ^+ 3.5SiO_2 _+ 5H_2_O		2.56
Kaolinite	Al_2_Si_2_O_5_(OH)_4 _+ 6 H^+ ^⇔ 2 Al^3+ ^+ 2 SiO_2 _+ 5 H_2_O		1.35
Magnesite	MgCO_3 _+ H^+ ^⇔ Mg^2+ ^+ HCO_3_^-^		0.71
Quartz	SiO_2 _⇔ SiO_2,aq_		-3.10
Siderite	FeCO_3 _+ H^+ ^⇔ Fe^2+ ^+ HCO_3_^-^		-1.40
Smectite (Ca-Beidellite)	Ca_0.165_Al_2.33_Si_3.67_O_10_(OH)_2 _+ 7.32H^+ ^⇔ + 0.165Ca^2+ ^+ 2.33Al^3+ ^+ 4.66H_2_O + 3.67SiO_2_		-0.62

Unless otherwise noted the values are estimated from SUPCRT92 [35]; ^1 ^values from CEMDATA [36].

**Table 6:** Rate parameters from Palandri and Kharaka [40].

Phase	Neutral		Acid			Base		
	k_25°C _(mol/m^2^/s)	E_a _(mol/KJ)	k_25°C _(mol/m^2^/s)	E_a _(mol/KJ)	N	k_25°C _(mol/m^2^/s)	E_a _(mol/KJ)	n
^a^Boehmite	-11.5	61.2	-7.7	47.5	0.99	-16.7	80.1	-0.78
Dolomite	-7.5	52.2	-3.2	36.1	0.50			
^b^Fe(OH)_3_								
Ripedolite	-12.5	88	-11.1	88.0	0.50			
Illite	-12.8	35	-11.0	23.6	0.34	-16.5	58.9	-0.40
Kaolinite	-13.2	22.2	-11.3	65.9	0.78	-17.0	17.9	-0.47
Low-albite	-12.6	69.8	-10.2	65.0	0.46	-15.6	71.0	-0.57
Quartz	-14.0	87.7						
Siderite	-8.9	62.76	-3.8	45.0	0.90			
Smectite	-12.8	35	-11.0	23.6	0.34	-16.5	58.9	-0.40

^a^Gibbsite dissolution rates were applied for boehmite. ^b^Fe(OH)_3 _rate was estimated from match to the solution chemistry normalized to mineral moles and is listed in Table 7.

**Table 7:** Conditional rate constants for the cement phases estimated from fits of the solution composition and anhydrous cement composition for each experiment.

Phase	Experiment	log k (mol/s/mol-mineral)
Anhydrite	^a^Cement:Brine	-6.2
	^b^Cement:Brine	-7.3
	Sandstone ± cement	-6.3
	Shale ± cement	-6.9
Hydrogarnet-Fe	^a^Cement:Brine	-6.2
	^b^Cement:Brine	-7.3
	Sandstone ± cement	-6.3
	Shale ± cement	-6.9
Hydrotalcite	^a^Cement:Brine	-5.5
	^b^Cement:Brine	-6.6
	Sandstone ± cement	-5.6
	Shale ± cement	-6.2
Brucite	^a^Cement:Brine	-5.5
	^b^Cement:Brine	-6.6
	Sandstone ± cement	-5.6
	Shale ± cement	-6.2
Portlandite	^a^Cement:Brine	-5.5
	^b^Cement:Brine	-6.6
	Sandstone ± cement	-5.6
	Shale ± cement	-6.2
Pseudowollastonite	^a^Cement:Brine	-6.2
	^b^Cement:Brine	-7.3
	Sandstone ± cement	-6.3
	Shale ± cement	-6.9
Amorphous Fe(OH)_3_	Sandstone	-8.6
Calcite	^b^Cement:Brine	-8.0
Calcite	Shale ± cement	-7.0
FeCO_3_	Shale ± cement	-10.0

The range in values reflects incomplete hydration and diffusion-controlled transport. ^a^Cement:Brine refers to experiment GBCO2_1 and ^b^Cement:Brine refers to experiment GBCO2_2.

All sandstone and shale reactions are assumed to be kinetically controlled and are modeled using

(1) $$r=\pm kA\left(1-\frac{LAP}{K_{sp}}\right)$$

where *r *is the dissolution or precipitation rate per unit time per unit area, *k *is the kinetic rate constant, *A *is the mineral surface area, *IAP *is the ion activity product, and *K_sp _*the solubility constant. Surface area is adjusted to fit the solution composition (Table 4). All of reactive surface area was assumed to be available for reaction for the sandstone experiments, whereas only 10 percent of the shale reactive surface area was assumed to participate in reactions. The rate constant for Fe(OH)_3 _precipitation in the sandstone experiments was fitted to the solution composition and is normalized the mineral moles (Table 7). All other rate constants are from Palandri and Kharaka [40] (Table 6):

(2) k=k25nu exp-EanuR1T-1298.15+k25H exp-EaHR1T-1298.15{H+}n

where *R *is the universal gas constant, *T *the absolute temperature, {*H*^+^} the activity of the hydrogen ion, *n *an exponential factor, and *k*_25_*^nu ^*and *k*_25_*^H ^*the neutral and acid rate constants at 25°C and *E*_a_*^nu ^*and *E*_a_*^H ^*the neutral and acid activation energies, respectively.

A few additional constraints to the geochemical model were needed to match Al, Fe, and Si solution chemistry and involve the precipitation of secondary phases. We added dissolved O_2 _to account for the attenuation of the initial spike in Fe in the sandstone experiment as secondary precipitation of Fe(OH)_3_. Quartz precipitation is suppressed and dissolved Si concentrations are limited by chalcedony equilibrium with no kinetic controls. Smectite precipitation is tied to illite surface area and is modeled as Ca-beidellite, Ca_0.165_Al_2.33_Si_3.67_O_10_(OH)_2_. Boehmite, AlO(OH), precipitation is suppressed until the solution is slightly supersaturated (log SI = 1). Uptake of Fe(II) during cement carbonation is modeled as an ideal continuous FeCO_3 _- CaCO_3 _solid solution. No other solid solutions are included in this model. We model cement carbonation from an assemblage of portlandite, psuedowollastonite, brucite, hydrotalcite, Fe-hydrogarnet, and anhydrite estimated from the hydration of initial anhydrous cement composition using the experimental cement to brine ratios (Table 2). We use pseudowollastonite to represent the amorphous hydrated calcium silicate ("CSH") because its solubility is consistent with our experimental measurements. Brucite and hydrotalcite were needed to account for the observed removal of Mg from the brine during cement hydration and its enhanced solubility during carbonation, even though these phases were not identified in the XRD pattern. Attempts to model the poorly crystalline phase as Mg-Ca silica hydrate introduced excessive amounts of dissolved silica. Anhydrite accounts for the sulfate noted in the anhydrous components.

Carbonation of the hydrated mineral assemblage was modeled with calcite, amorphous SiO_2 _(as chalcedony), Fe(OH) _3_, and boehmite for cement: brine < 1:50 or amorphous Al(OH)_3 _for cement: brine > 1:25 (Table 2). Fitted rate constants for the cement phases reflect the varied extent of cement hydration and carbonation in each experiment and are normalized to mineral moles (Table 7). It is important to note that the fitted reaction rates are conditional to these experiments and likely represent diffusion of the reactants into the cement, which had a tendency to solidify at the bottom of the reaction vessel even though the experiments were rocked.

#### Model Uncertainty

It is important to note that the lithology and cement geochemical models represent possible realizations of the dominant geochemical reactions. The non-uniqueness of the lithology model reflects the wide range of minerals that can be used to describe major element chemistry and was made apparent in our efforts to fit the dissolved Si prior to the injection of CO_2 _in the sandstone experiment. The best matches required either using unrealistically high illite in the sandstone (higher than in the shale) or alternatively excessive high quartz surface areas (which were also higher than in the shale). Presumably, some combination of aluminosilicate dissolution and enhanced quartz dissolution (in the sandstone) is responsible for the Si accumulation prior to CO_2 _injection. Compositional variations among carbonates, chlorite, and illite could not be considered owing to a lack of thermodynamic data. Additionally, phases such as boehmite or Fe(OH)_3 _represent idealizations of aluminum- or ferric oxyhydroxides that may, in reality, may be characterized by different stoichiometries or crystal structure than those phases represented in the thermodynamic database.

A significant number of factors contribute to the uncertainty in the cement carbonation geochemical model. Mineralogy, including the pertinent stoichiometry and equilibrium constants, of the hydrated cement is poorly constrained by the experimental data. Moreover, the mineralogical sink for magnesium is unconstrained and may or may not be associated with the CSH phase. Solid solutions may be important for a number of phases in the hydrated cement and carbonated mineral datasets as well as in the reservoir lithology. We employ either pure phase or ideal solid solution because data to constrain non-ideal solid solutions are lacking.

## Experimental Results

### Cement Hydration at 95 and 115°C

**Figure 1:**
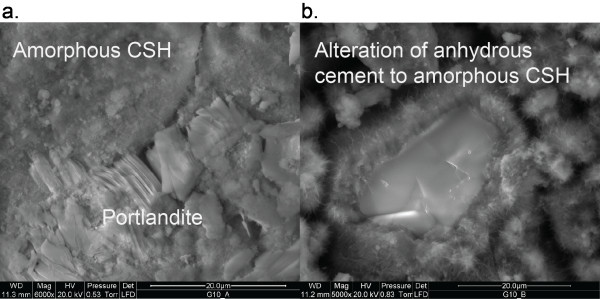
**ESEM images of Class G cement reacted in 0.13 molal CaCl_2 _solution showing crystalline portlandite, residual anhydrous Ca_2_SiO_4 _and amorphous CSH**.

Cement hydration with reservoir brines altered both the brine chemistry and the hydrated cement phases in experiments at 95 and 115°C (Table 8). Our results show that when Class G cement reacts with distilled water or 0.13 molal CaCl_2 _brine, the water composition is largely controlled by the solubility of portlandite and an amorphous calcium silica hydrate (CSH). Portlandite was identified as the only crystalline phase by XRD. ESEM analysis also showed crystalline portlandite, as well as an amorphous calcium silicate, which we assume to be CSH (Figure 1).

**Table 8:** Measured solution composition for cement hydration and the experiment end.

ID	Al molal	Ca molal	Fe molal	Mg molal	Si molal	F^-^ molal	Cl^-^ molal	NO_3_^-^ molal	SO_4_^2-^ molal
G3	1.39E-05	0.109	3.68E-07	ND	1.73E-05				
G6	1.91E-05	4.68E-01	9.64E-07	1.34E-05	4.87E-06				
G7	1.70E-05	7.83E-03	1.07E-07	ND	8.53E-06	5.86E-05	1.73E-05	2.38E-05	1.81E-03
G8	1.89E-05	6.62E-03	2.21E-07	ND	3.99E-05	6.01E-05	1.66E-04	2.21E-05	1.77E-03
G9	3.30E-05	4.42E-03	6.30E-08	ND	9.89E-06	8.06E-05	1.54E-05	2.91E-05	3.64E-03
G10	2.76E-05	6.01E-03	7.99E-08	ND	3.44E-05	1.21E-04	2.30E-05	2.60E-05	3.85E-03
G11	2.05E-05	7.82E-03	7.30E-08	ND	3.30E-05	5.51E-05	2.42E-05	2.43E-05	2.33E-03
G12	4.22E-05	5.74E-03	6.89E-08	ND	8.56E-06	7.06E-05	1.93E-05	2.59E-05	4.10E-03
G13	3.12E-05	5.90E-03	7.43E-08	ND	5.31E-05	6.96E-05	3.89E-05	2.77E-05	4.85E-03
G14	3.05E-06	5.88E-01	1.46E-06	5.41E-06	1.89E-07		2.97		
G15	3.35E-06	5.82E-01	1.38E-06	5.95E-06	2.05E-07		3.00		

Blank values indicate that the ions were not measured. ND indicates concentrations were below detection.

The storage reservoir brine is likely to be distinct from the dilute waters used to mix the cement prior to injection in the well. The experiments in synthetic brine show that mineral assemblage at the cement - brine interface will be different than in the interior of the cement. The primary difference is that Mg has a very low solubility when the brine reacts with the anhydrous cement. Cement hydration produced a less alkaline and Mg-poor brine (Mg = 10^-5 ^molal). Although the solution was supersaturated with respect to brucite, Mg(OH)_2_, the resulting Mg-bearing phase was poorly crystalline and could not be identified by XRD.

There is a large amount of uncertainty associated with the identification of stable or metastable cement minerals in the wellbore environment, because cements can range in composition and are often amorphous. Possible cement phases include anhydrous belite (Ca_2_SiO_4_) which was present as a residual reactant in most of the experiments, and CSH phases such as hillebrandite (Ca_2_SiO_3_(OH)_2_*0.17H_2_O), jennite (Ca_1.67_SiO_2_(OH)_3.33_: 0.43H_2_O), and tobermorite-CSH (Ca_0.83_SiO_2_(OH)_1.7_*0.5H_2_O). Average ion activity products calculated from the solution chemistry at 95 and 115°C are listed in Table 9. It is likely that cements will alter to crystalline phases with time, because the transformation from amorphous to stable phases has been observed at 150°C for tobermorite [41]. Given the amorphous nature of CSH in our experiments, we have chosen to model it as psuedowollastonite (CaSiO_3_) because measured solution compositions are near psuedowollastonite equilibrium (Table 5).

**Table 9:** Calculated ion activity products (IAP) for select CSH phases

Cement Hydration			
Phase	Mass Balance	Log IAP 95°C	Log IAP 115°C
Belite	Ca_2_SiO_4 _+ 4H^+ ^= 2Ca^2+ ^+ SiO_2_(aq) + 2H_2_O	29.6 ± 1.2	28.9 ± 0.4
Hillibrandite	Ca_2_SiO_3_(OH) _2_*0.2H_2_O + 4H^+ ^= 2Ca^2+ ^+ SiO_2_(aq) + 3.2H_2_O	29.6 ± 1.2	28.9 ± 0.4
Jennite - CSH	Ca_1.67_SiO_2_(OH)_3.33_: 0.43H_2_O + 3.33H^+ ^= 1.67Ca^2+ ^+ SiO_2_(aq) +3.76H_2_O	27.8 ± 1.4	27.3 ± 0.5
Tobermorite	Ca_0.83_SiO_2_(OH)_1.7_*0.5H_2_O + 1.66H^+ ^= 0.83Ca2+ + SiO_2_(aq) + 2.2H_2_O	11.7 ± 0.7	11.1 ± 0.5
Pseudowollastonite	CaSiO_3_+ 2H^+ ^⇔ SiO_2 _+ Ca^2+ ^+ H_2_O	11.3 ± 1.3	11.5 ± 0.5

### Cement - Rock - Brine - CO_2 _Experiments

In this section we describe the experimental results from the reaction of supercritical CO_2 _and synthetic brine with reservoir rock, cap rock, and wellbore cement. The sandstone and shale used in the experiments are meant to represent storage reservoir and the cap rock respectively (although the materials themselves are from the heterolithic sandstone and shale unit that forms the upper section of the carbon storage reservoir at the Krechba Field, In Salah, Algeria). Interpretation of the rate and solubility controlling reactions will be discussed in Geochemical Model.

#### Sandstone - brine - CO_2_

**Figure 2:**
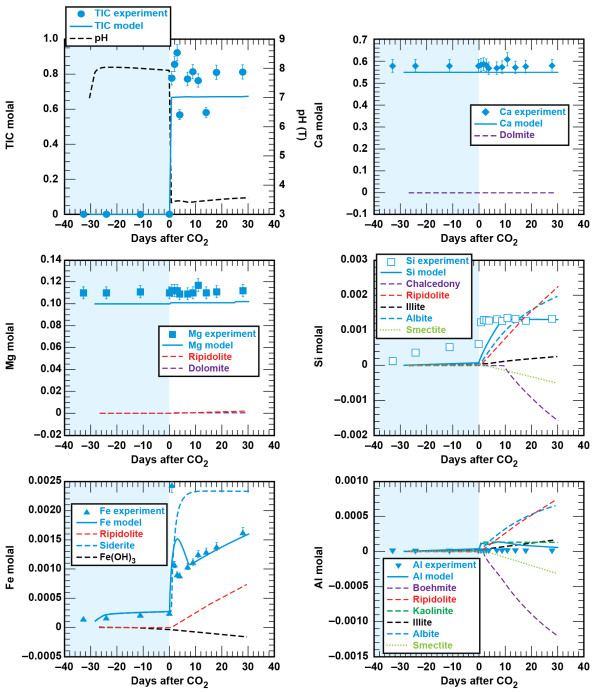
**Carbonation of sandstone plotted as solution composition versus reaction time**. Lines are the modeled results.

**Figure 3:**
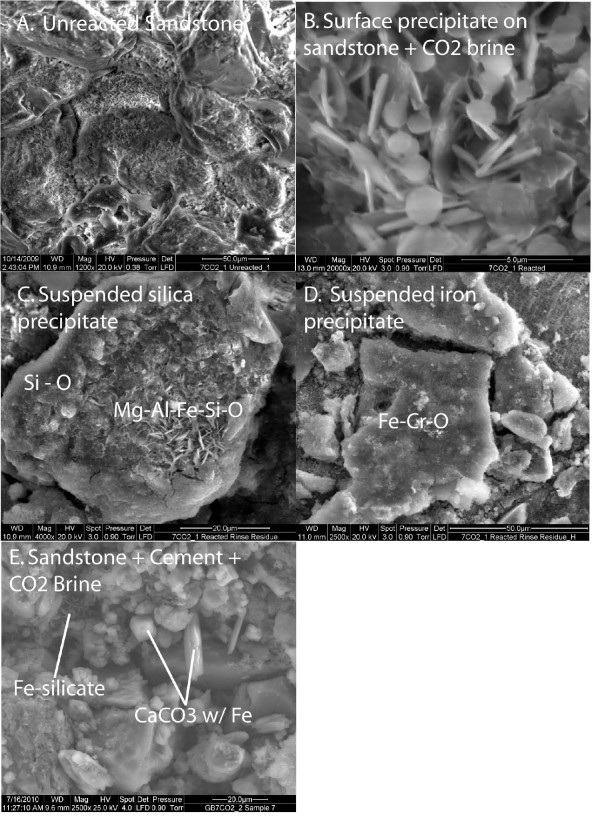
**ESEM images of reacted sandstone.** (A) Unreacted sandstone consisted of quartz, carbonate cement, and chlorite which lines the pore spaces. Reaction of sandstone with CO_2_-rich brine produced (B) aluminum hydroxide or aluminosilicate reaction products deposited on the sandstone surface and (C-D) coagulated Si-rich and Fe-rich precipitates in the brine. Reaction of the sandstone with cement and CO_2_-rich brine produced (E) Fe - bearing CaCO_3 _precipitates.

The sandstone consists of tightly carbonate-cemented quartz grains with about 7 wt % chlorite lining the pores [33]. Figures 2 and 3 show the solution profiles with time and images of the unreacted and reacted sandstone. Reaction of sandstone with brine produced a fairly neutral solution with low dissolved CO_2_, dissolved Ca and Mg near the initial brine concentrations, dissolved Si and Fe that increased slowly with time, and very low dissolved Al. Injection of supercritical CO_2 _into the reaction vessel resulted in marked increases in dissolved CO_2_, Si, and Fe. Dissolved CO_2 _and Si increase rapidly to a constant concentration. Dissolved Fe peaked upon injection of CO_2_, dropped to a minimum value and then increased linearly with time. No abrupt changes were observed in dissolved Ca, Mg, or Al.

Secondary precipitates were either amorphous to XRD or present in amounts below the XRD detection limit for crystalline phases to be identified. ESEM images show relatively large amounts of silica and iron precipitates as well as thin hexagonal sheet silicates (Figure 3). It is not possible to identify the composition with EDX because the beam samples an area larger than the surface precipitates. It is also possible that the precipitates formed as the solution was cooled prior to taking apart the reaction vessel and recovering the solids for analysis.

#### Shale - brine - CO_2_

**Figure 4:**
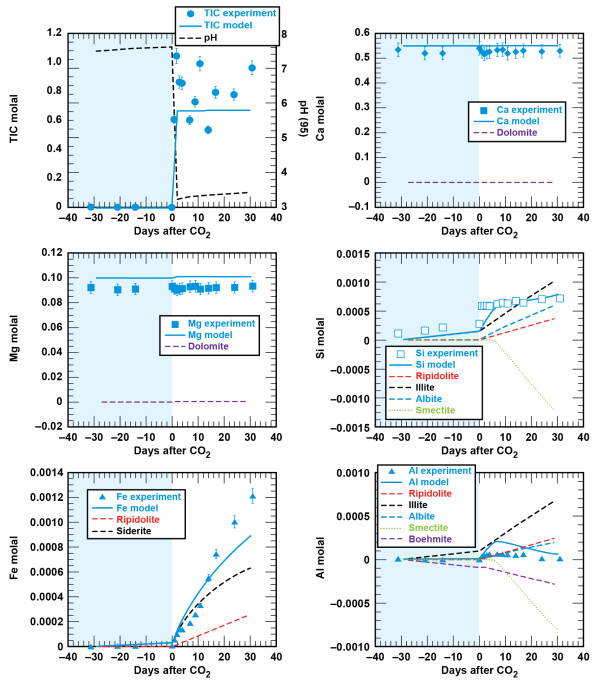
**Carbonation of shale plotted as solution composition versus reaction time**. Lines are the modeled results.

**Figure 5:**
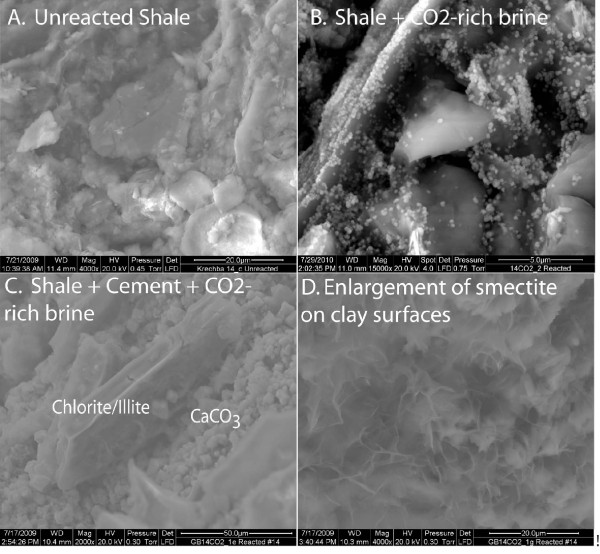
**ESEM images of reacted shale.** (A) Unreacted shale consisted of fine-grained quartz, illite, and carbonate. When the shale was reacted with CO_2_-rich brine (B) submicron reaction products deposited on the shale surface and in solution. When the shale reacted with cement and the CO_2_-rich brine (C-D) there was extensive clay dissolution and precipitation of smectite and calcium carbonate.

Figures 4 and 5 show the solution profiles with time and images of the unreacted and reacted shale. Similar to the sandstone experiment, reaction of shale with brine produced a fairly neutral solution with low dissolved CO_2_, dissolved Ca and Mg near the initial brine composition, dissolved Si that increased slowly, and very low dissolved Fe and Al. Injection of supercritical CO_2 _into the reaction vessel produced a marked increase in dissolved CO_2_, Si, Fe, and Al, with no change in the dissolved Ca and Mg.

We see very little indication of alteration of the shale by CO_2_-rich brines at the experiment end by ESEM/EDX analysis (Figure 5). We detect only small precipitates on the shale surface and in the suspension, which may have formed when the sample was quenched from 95°C to room temperature. Particle size was too small to confirm the chemical composition with EDX.

#### Cement - brine - CO_2_

**Figure 6:**
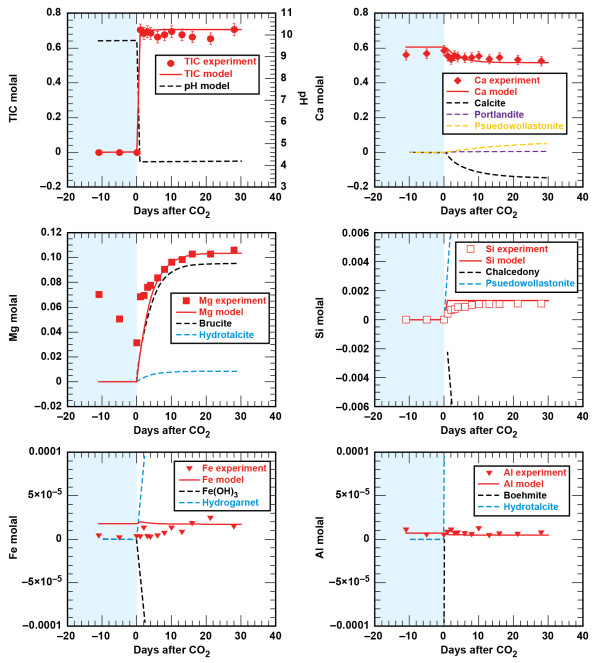
**Carbonation of class G cement as solution composition versus reaction time with a solid(g): brine(g) ≈ 1:68**. Lines are the modeled results.

**Figure 7:**
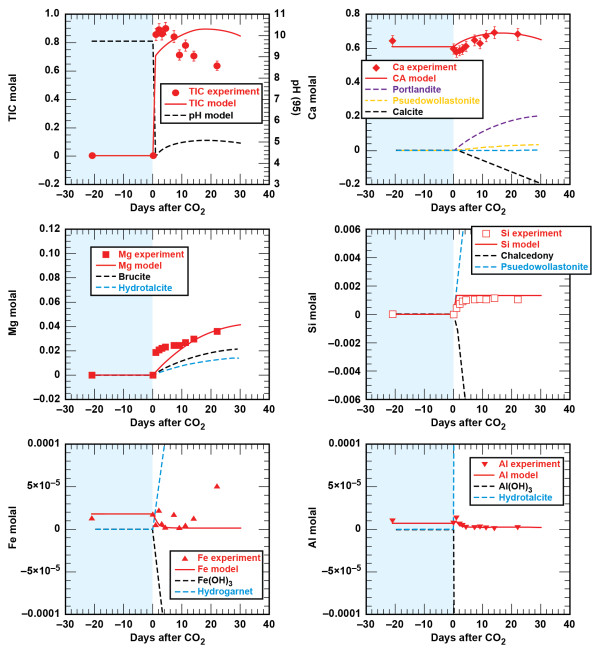
**Carbonation of class G cement as solution composition versus reaction time with a solid(g): brine(g) ≈ 1:10**. Lines are the modeled results.

Cement altered to aragonite, calcite, and amorphous silica by the CO_2_-rich brines. XRD analyses show aragonite, calcite, and residual anhydrous Ca_2_SiO_3_. We assume that Si from the CSH phase was altered to amorphous silica. Figures 6 and 7 show the change in solution composition for the reaction of cement, brine, and supercritical CO_2 _at 95°C and 10 MPa as solution pH (95°C), total dissolved CO_2_, Ca, Mg, Si, Fe, and Al. These experiments had solid to brine ratios of 1:68 and 1:10 on g/g basis. Trends in dissolved Ca and Mg suggest that starting materials may not have been fully hydrated prior to the injection of CO_2_. Extrapolation of the linear decrease in Mg to values measured in the solubility experiments, suggest that the cements would fully equilibrate with the brine within 20 days of reaction at 95°C. The lack of complete hydration is of little consequence because the cement system is very reactive in brines with supercritical CO_2_. Upon injection of supercritical CO_2_, there was a marked increase in dissolved CO_2, _dissolved Ca decreased, dissolved Mg increased toward their initial brine concentration, dissolved Si increased to a constant value, and dissolved Fe and Al were quite low.

Experiments with higher amounts of cement resulted in solidification of cement at the bottom of the reactor. We suspect that dissolution was ultimately limited by diffusion at the cement-solution interface. Less than 40% of the cement reacted with CO_2_-rich brine based on the dissolved Mg. We estimate the extent of the cement carbonation reaction from the recovery of Mg in solution, because the brines are undersaturated with respect to magnesite (MgCO_3_) and there is no indication of Mg in the carbonate precipitates. Another difference between this experiment and the one at lower cement: brine was that the dissolved Ca increased with time.

#### Cement - sandstone - brine - CO_2_

**Figure 8:**
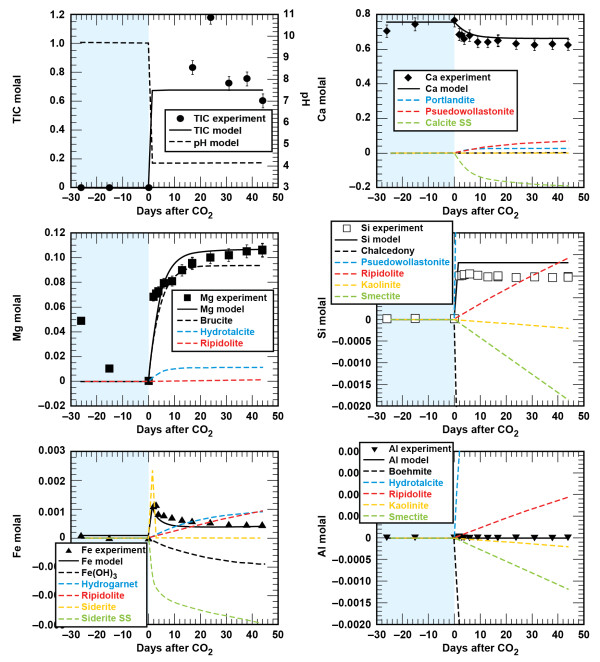
**Carbonation of class G cement and sandstone plotted as solution composition versus reaction time**. Lines are the modeled results.

Comparison of the solution chemistry profiles from the cement and cement - sandstone experiments suggest that cement carbonation will drive reaction chemistry in the wellbore environment where the cement contacts sandstone geology (Figures 2 and 8). In the first phase of the experiment, cement hydration produced alkaline solutions with elevated Ca and depleted Mg. The cement mineral assemblage underwent rapid carbonation when supercritical CO_2 _was injected into the brine. Dissolved CO_2 _increased by several orders of magnitude to values between 0.6 and 0.8 molal, dissolved Ca decreased, dissolved Mg increased to the initial brine concentration, dissolved Si also increased to a constant value, and dissolved Al was quite low.

Despite the dominance for the cement carbonation reactions, there is a chemical signature from the sandstone. Upon injection of the CO_2_, the dissolved Fe increased by 3 orders of magnitude to a peak concentration, and then decreased over time to a constant value. This is in sharp contrast to the continued increase in dissolved Fe when sandstone was reacted with CO_2_-rich brines in the absence of cement. Qualitative EDX analyses show some Fe in rhombahedral and bladed shaped calcium carbonate alteration products (Figure 3). Fe was also detected in the thin bladed micron-sized silicates that are either residual chlorite or a secondary smectite or iron hydroxide. Cr is also detected in these micron-sized crystals.

#### Cement - shale - brine - CO_2_

**Figure 9:**
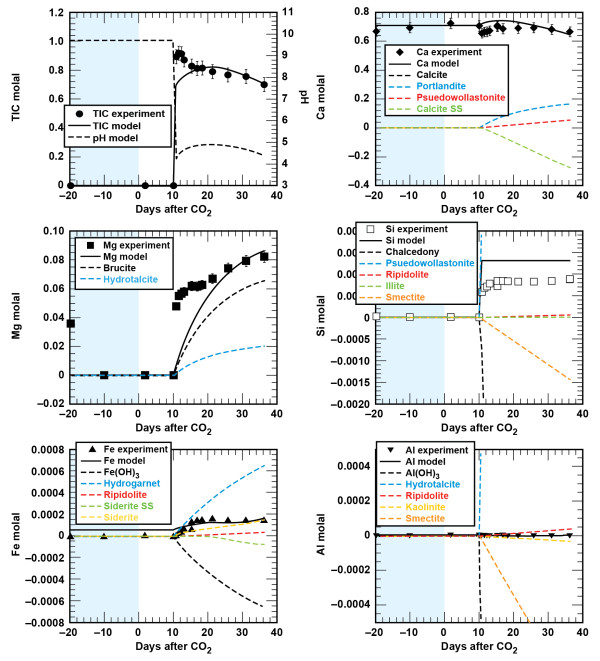
**Carbonation of class G cement and shale plotted as solution composition versus reaction time**. Lines are the modeled results.

Similar to cement - sandstone - CO_2 _experiment, the cement carbonation chemistry drives the dominant alteration products when shale is reacted with cement and CO_2_-rich brines (Figure 9). Cement hydration in this experiment was analogous to the other experiments, producing an alkaline solution with elevated Ca and depleted Mg. Reaction of supercritical CO_2_, brine, shale and cement yielded Mg, Si, and Fe profiles that are different from their respective profiles in the cement and cement - sandstone experiments. About 80% of the Mg removed from the brine during the cement hydration phase of the experiment was recovered in the solution at the experiment end suggesting that 80% of the bulk cement was carbonated. Dissolved Si increased to a level below that observed for experiments with cement and cement - sandstone. Dissolved Fe approached a value similar to the final concentrations measured in the cement - sandstone experiments, and dissolved Al was quite low.

SEM images show that sheet silicates were altered when the shale reacts with the cement and CO_2_-rich brines (Figure 5). Extensive dissolution groves formed along the edges of the sheet silicates and fibrous precipitates formed on the planar surfaces of the sheet silicates, in addition to calcium carbonate precipitation from cement carbonation.

## Geochemical Model

Geochemical modeling was used to identify a plausible *set of reactions *consistent from (1) the reported mineralogy from the Krechba reservoir, (2) the changes in brine chemistry observed during each of the experiments, and (3) the alteration products identified at the end of the experiments. Our objective in creating the geochemical model was to preserve key attributes, such as mineral composition and dissolution rates, across all experiments to better constrain conceptual models for the assessment of long-term CO_2 _trapping mechanisms and wellbore and cap rock integrity using reactive-transport simulations. It is important to note that the geochemical model represents one realization that describes six experiments. Details of the modeling approach and related uncertainties can be found in Modeling Uncertainty.

### Sandstone Reservoir and Shale Cap Rock Geochemical Model

The geochemical model for the reaction of sandstone and shale with CO_2 _and brine is a simple one, in which chlorite, illite, albite, quartz and carbonate minerals partially dissolve and boehmite, smectite, Fe(OH)_3 _and amorphous silica precipitate (Table 5). The same geochemical model is used to describe both the storage reservoir and the cap rock, because the mineralogy is the same for both rock types, although the relative proportion of the minerals differs.

Comparison of the measured and simulated data shows that this simple model adequately describes the experiments (Figures 2 and 4). In both experiments dolomite dissolution predicts dissolved Ca and Mg to within 5% of the experimental values. Upon injection of CO_2 _into the sandstone experiment dissolved Si from albite, chlorite, and lesser amounts of illite dissolution is offset by silica precipitation once chalcedony saturation is exceeded and some smectite precipitation. In the sandstone experiment small amounts of illite limit the amount of smectite precipitation, which is tied to the illite surface area. However, in the shale experiment, where there is significantly more illite (44%) than in the sandstone experiment (1.5%), smectite precipitation effectively limits the dissolved Si to concentrations below chalcedony saturation. Pre-CO_2 _and the abrupt changes in dissolved Si, possibly from the dissolution of fines that occurred when CO_2 _was first injected into the sandstone experiment, are not captured in the model fits. Both siderite and chlorite dissolution contribute to the dissolved Fe concentrations in both experiments. The sharp decrease in dissolved Fe in the sandstone experiment can be modeled as Fe(OH)_3 _precipitation and the depletion of dissolved oxygen present in the stock solutions that were prepared at atmospheric conditions. Any dissolved oxygen appears to have been quickly consumed in the shale experiment as no concentration peaks were observed. The low dissolved aluminum concentrations are a product of secondary precipitation of boehmite, kaolinite, and smectite.

### Wellbore Geochemical Model

We derive the wellbore geochemical model by combining the lithologic model with cement hydration and carbonation models described below. Carbonation of the hydrated cement assemblage was modeled with a set of carbonate minerals, amorphous SiO_2 _(as chalcedony), Fe(OH)_3_, and boehmite or amorphous Al(OH)_3 _(dependent on the cement: brine; Table 5, Figures 6 and 7). Comparison of the measured and simulated data shows that this simple model adequately describes the data and captures the effects of reacting varying amounts of cement with the CO_2_-rich brine. At low solid to brine ratios (1:68 g/g), calcite precipitation results in a decrease in dissolved Ca, brucite and hydrotalcite dissolution result in the recovery of dissolved Mg to initial values, and SiO_2_, Fe(OH)_3 _and boehmite precipitation limit the amount of dissolved Si, Fe, and Al as CSH, Fe-hydrogarnet and hydrotalcite dissolved during the carbonation process. At higher solid to brine ratios (1:10 g/g), where roughly 60% more cement reacted with brine (based on percent recovery of dissolved Mg and the initial amount of cement), the model captures the increase in dissolved Ca with cement carbonation and higher dissolved Al concentrations when amorphous Al(OH)_3 _is used to control Al solubility. Recall, that rates used here are conditional to the experiments and scale with dissolved Mg recovery.

The combination of the lithology - brine - CO_2 _and cement carbonation models reproduces brine chemistry evolution observed during the carbonation phases of the composite experiments (Figures 8 and 9). As might be expected, cement carbonation dominates the geochemical reactions in the wellbore environment, largely because cement reactivity masks contributions from the much less reactive sandstone and shale minerals. Dissolved Ca can be accounted for by the carbonation of portlandite and CSH. Dissolved Mg can be accounted for by dissolution of brucite and hydrotalcite (where the extent of cement carbonation is fit to the proportion of Mg recovered). Although chalcedony precipitation accounts the bulk of Si during carbonation, the higher inputs of Si and Ca result in smectite precipitation in both the cement - sandstone and cement - shale experiments. This model result agrees with the appreciable amount of smectite observed in the cement - shale experiment. One added parameter specific to the lithology - cement - brine - CO_2 _experiments was the introduction of a ferroan calcite solid solution, which limited the dissolved Fe from chlorite dissolution in the sandstone and shale experiments.

## Conclusions

Our research shows that relatively simple geochemical models can describe the dominant reactions that will occur when CO_2 _is stored in deep saline aquifers sealed with overlying shale cap rocks, and when CO_2 _reacts at the interface between cement and reservoir and shale cap rock. Although the experiments and modeling reported here are specific to the CO_2 _storage at the Krechba site, the model may be applicable to other storage sites with similar geology. Development of these relatively simple geochemical models is needed to assess long-term CO_2 _trapping mechanisms, cap rock and wellbore integrity in more computationally intensive reactive-transport simulations that couple chemistry, flow, and possibly geomechanics. As is expected, Al/Fe silicate dissolution drives the geochemical alterations within the reservoir and cap rock pore space. Addition of CO_2 _lowers the pH and promotes silicate dissolution and amorphous silica, smectite and boehmite precipitation. The dissolved Fe may be a source of long-term mineral trapping of CO_2 _and the precipitation of secondary Fe-carbonates, clays and hydroxides could alter reservoir and seal permeability by clogging pores and fracture networks. In agreement with other studies we find that alkaline cements are highly reactive in the presence of CO_2_-rich brines and are quickly transformed to carbonate minerals and amorphous silica. These reactions can be easily modeled as the transformation of portlandite, and Ca- and Mg-silicates to aragonite or calcite and amorphous silica. Finally, we find that dissolved Mg common in deep saline brines will react with the wellbore cement to form poorly-crystalline solids. Additional research is required to assess mineral structure of the Mg-rich cement phase, as it could not be identified in this study and to assess what the impact of the Mg - induced alteration may have on wellbore integrity.

## Competing interests

The authors declare that they have no competing interests.

## Authors' contributions

SAC is the primary author. She designed and directed the experiments, WWM modeled the experiments, and SCT conducted the experiments. All authors have read and approved the final manuscript.
